# Overcoming Depression on the Internet (ODIN) (2): A Randomized Trial of a Self-Help Depression Skills Program With Reminders

**DOI:** 10.2196/jmir.7.2.e16

**Published:** 2005-06-21

**Authors:** Greg Clarke, Donna Eubanks, Ed Reid, Chris Kelleher, Elizabeth O'Connor, Lynn L DeBar, Frances Lynch, Sonia Nunley, Christina Gullion

**Affiliations:** ^1^Kaiser Permanente Center for Health ResearchPortland, ORUSA

**Keywords:** Internet, depression, cognitive therapy, self-help, randomized trial

## Abstract

**Background:**

Guided self-help programs for depression (with associated therapist contact) have been successfully delivered over the Internet. However, previous trials of pure self-help Internet programs for depression (without therapist contact), including an earlier trial conducted by us, have failed to yield positive results. We hypothesized that methods to increase participant usage of the intervention, such as postcard or telephone reminders, might result in significant effects on depression.

**Objectives:**

This paper presents a second randomized trial of a pure self-help Internet site, ODIN (Overcoming Depression on the InterNet), for adults with self-reported depression. We hypothesized that frequently reminded participants receiving the Internet program would report greater reduction in depression symptoms and greater improvements in mental and physical health functioning than a comparison group with usual treatment and no access to ODIN.

**Methods:**

This was a three-arm randomized control trial with a usual treatment control group and two ODIN intervention groups receiving reminders through postcards or brief telephone calls. The setting was a nonprofit health maintenance organization (HMO). We mailed recruitment brochures by US post to two groups: adults (n = 6030) who received depression medication or psychotherapy in the previous 30 days, and an age- and gender-matched group of adults (n = 6021) who did not receive such services. At enrollment and at 5-, 10- and 16-weeks follow-up, participants were reminded by email (and telephone, if nonresponsive) to complete online versions of the Center for Epidemiological Studies Depression Scale (CES-D) and the Short Form 12 (SF-12). We also recorded participant HMO health care services utilization in the 12 months following study enrollment.

**Results:**

Out of a recruitment pool of 12051 approached subjects, 255 persons accessed the Internet enrollment site, completed the online consent form, and were randomized to one of the three groups: (1) treatment as usual control group without access to the ODIN website (n = 100), (2) ODIN program group with postcard reminders (n = 75), and (3) ODIN program group with telephone reminders (n = 80). Across all groups, follow-up completion rates were 64% (n = 164) at 5 weeks, 68% (n = 173) at 10 weeks, and 66% (n = 169) at 16 weeks. In an intention-to-treat analysis, intervention participants reported greater reductions in depression compared to the control group (*P* = .03; effect size = 0.277 standard deviation units). A more pronounced effect was detected among participants who were more severely depressed at baseline (*P* = .02; effect size = 0.537 standard deviation units). By the end of the study, 20% more intervention participants moved from the disordered to normal range on the CES-D. We found no difference between the two intervention groups with different reminders in outcomes measures or in frequency of log-ons. We also found no significant intervention effects on the SF-12 or health care services.

**Conclusions:**

In contrast to our earlier trial, in which participants were not reminded to use ODIN, in this trial we found a positive effect of the ODIN intervention compared to the control group. Future studies should address limitations of this trial, including relatively low enrollment and follow-up completion rates, and a restricted number of outcome measures. However, the low incremental costs of delivering this Internet program makes it feasible to offer this type of program to large populations with widespread Internet access.

## Introduction

Several Internet interventions have emerged in recent years to treat mental and behavioral health problems. These interventions provide some of the basic skills training traditionally offered in face-to-face psychotherapies, particularly cognitive behavioral therapy (CBT). This recent trend extends the tradition of bibliotherapy with books, videos [1–3], and computer programs [4]. Mental health Internet interventions have targeted panic disorder [5,6], distress associated with tinnitus [7], and depression [8,9]. Nearly all of these “guided self-help” interventions [10] incorporate the Internet skills training with simultaneous professional staff counseling typically delivered by telephone or email.

Our Internet program, ODIN (Overcoming Depression on the InterNet) [11], shares a CBT approach with these other interventions. However, it is “pure self-help” [10] because it relies solely on skills training delivered by the Internet and eschews the therapist-delivered mental health counseling typical of the other programs. Both guided and pure self-help approaches merit consideration, but the much lower cost of the latter is a significant advantage.

Several of these interventions have been evaluated in randomized trials, with generally positive results on depression symptomatology for *guided* self-help programs [5,7,8]. However, initial trials of *pure* self-help Internet programs failed to impact depression symptoms [9], including our first investigation of the ODIN program [11]. In this earlier study, we randomized 299 adults with highly elevated depression symptoms to either access to the ODIN site, or no access. Participants in both conditions were free to receive treatment as usual (TAU) health care services, including depression medication and psychotherapy. This TAU control condition, consisting principally of antidepressant medication, distinguishes our research from that of most other trials of Internet mental health interventions, which have employed a waitlist control condition. Subjects reported depression symptoms at enrollment and at 4-, 8-, 16-, and 32-weeks follow-up. However, in that trial we found that participants in the intervention group used the ODIN Internet site very infrequently after their initial enrollment session, which may have contributed to the overall negative effects. We concluded that future studies should focus on increasing participant use of the Internet site.

This paper presents the second trial of our pure self-help ODIN program. This time, we added telephone and postcard reminders to the intervention group aimed at increasing participant use of ODIN, and we compared the intervention against a “no access” TAU control condition. We had no hypotheses regarding different website usage attributable to postcard or telephone reminders. However, the latter method required so much more staff time that we wanted to test whether brief telephone contact increased website usage beyond the less expensive postcard reminder. We hypothesized that persons randomized to the ODIN group would report greater reductions in depression symptoms and greater improvements in mental and physical health functioning. We also report general medical and mental health care service utilization data of participants in the 12 months following randomization.

## Methods

### Subjects and Recruitment

We conducted the study in the Kaiser Permanente Northwest HMO, which has about 440000 members in northwest Oregon and southwest Washington. Our research center is located within the HMO and is scientifically autonomous. The Human Subjects Committee for the HMO approved study procedures.

We employed the HMO's electronic medical record to identify two recruitment groups in 2000: a “depressed” group of adults (n = 6030), who received depression medication or psychotherapy in the previous 30 days and had a chart diagnosis of depression; and a “nondepressed” group of adults (n = 6021), who did not receive such services and did not have an HMO diagnosis of depression but who was age and gender matched to the first group. We included the latter group to determine whether persons with previously undetected cases of depression might enroll in the study.

We mailed all potential participants a study recruitment brochure in a plain envelope. The brochure explained the study and provided the Internet address. It was up to the initiative of invited individuals to visit the study Internet site.

**Figure 1:**
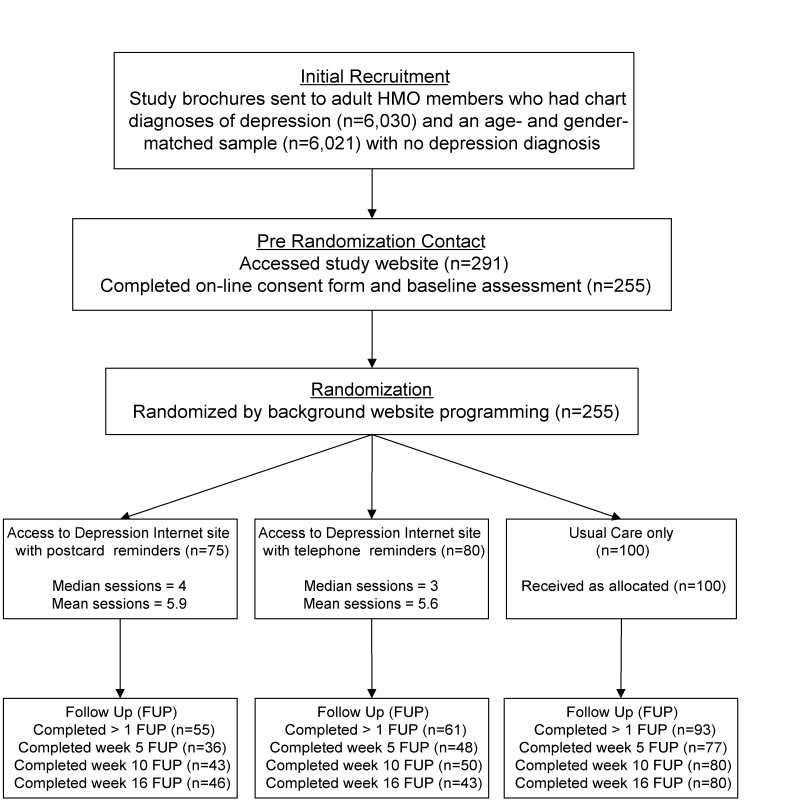
Study flowchart

After receiving the study recruitment brochure, participants entered confirmed HMO membership numbers at the study home page and proceeded to the online consent form and baseline assessment battery. Subsequently, participants were automatically randomized by the website (using random sequence software) to one of the three groups. Participants in the TAU control group were denied access to the ODIN intervention. Instead, they were linked to an HMO health information website which provided information about depression but no interactive skills training. Participants in the remaining two intervention groups were given immediate access to the ODIN intervention and received either US mail postcards or brief (< 5 minutes) telephone reminders from non-clinician study staff at 2, 8, and 13 weeks after enrollment. The telephone reminder calls were scripted to convey information identical to that included on the postcard reminders. Staff first identified themselves and the study, then reminded participants of the ODIN website address and gave instructions for looking up forgotten passwords. They read a brief description of a feature of the website designed to entice the participant to make a return visit and then concluded the call. The reminder staff had no mental health background, and they were prohibited from engaging in any therapy-like activity. Staff were capable of, and limited to, answering questions only about basic website troubleshooting (eg, difficulty logging on or accessing questionnaires). Figure 1 provides a summary of the study process.

Participants in all conditions were free to obtain any traditional mental or physical health care services and access any Internet health resources. Participants were not blind to their study condition.

### Assessment Battery

At baseline and at each follow-up, participants completed an online version of the Center for Epidemiological Studies Depression Scale (CES-D) [12], a self-report measure of 20 depressive symptoms. Participants also completed the Short Form 12 (SF-12), a measure of health-related functioning [13,14]. A Physical Component Summary (PCS) scale and a Mental Component Summary (MCS) scale were computed from the SF-12 items [15].

Computerized depression instruments generally yield psychometrics equivalent to paper versions [16]; both versions of the CES-D correlate highly (*r* = .96) [17]. Patients often prefer computerized methods for reporting sensitive health topics [16].

Subjects in all conditions were sent email reminders to complete the online follow-up questionnaires at 5, 10, and 16 weeks after enrollment. Study staff telephoned participants who failed to respond to two email reminders for any assessment. Participants received US $5, $10, $15, and $20 gift certificates to Amazon.com for completing the baseline and subsequent assessments.

### Intervention

The ODIN Internet intervention (www.feelbetter.org) was a pure self-help program offering training in cognitive restructuring [18,19]. (See the Multimedia Appendix of our previous report [11] for screenshots.) We did not employ any behavioral therapy or behavioral activation techniques. Intervention content was adapted from CBT psychotherapy manuals [20,21] successfully employed in randomized trials [22–25]. The intervention was organized in seven “chapters,” each presenting a new technique via interactive examples and practice opportunities. Tutorials included the self-assessment of mood, identification of unrealistic thoughts, and generation of realistic counter-thoughts. Participants randomized to the intervention conditions were able to use the program at any time.

A representative module was the “Thought Helper.” Participants typed their personal negative or irrational thought into a text box and then clicked on a search button. The Web server searched a predefined list of 300 negative thoughts for examples that best matched the negative thought submitted by the participant and returned a list of the most likely matches. Participants selected the displayed negative thought that they thought was closest to their original. The program then returned a list of several possible realistic counter-thoughts relevant to that belief. Users were encouraged to create a personalized counter-thought using relevant portions of the provided examples and enter it into the website for storage. Users could later retrieve their own personal counter-thoughts, unrealistic beliefs, and activating situations.

We did not actively monitor the participant interactions for suicidal thoughts or behaviors, but instead provided links to the non-research HMO psychiatric emergency services staffed by professional mental health providers.

### Health Care Utilization

HMO computer systems provided data for inpatient and outpatient services, prescriptions, emergency room visits, and other utilization. Non-HMO health care services were not assessed.

### Analysis Plan

We examined CES-D and SF-12 scores using random effects regression analyses, modeling an unstructured covariance matrix, with slope and intercept as random effects. The test of difference between groups is a test of the difference in these slopes over time. The random effects modeling includes all data on all participants (an intent-to-treat analysis), but it preserves the measurement time for each observed response (rather than carrying last observations forward). It does so by computing maximum likelihood estimates of the slope over time given the data observed and the covariance structure within subjects. This method, which conditions out the missing data, is called restricted (or residual) maximum likelihood estimation (REML). The REML methods for dealing with missing data are superior in efficiency and are considered less biased than the last observations carried forward (LOCF) method [26,27]. For all outcomes analyses (except for health care utilization), we conducted planned comparisons of (a) the two intervention conditions combined versus the control condition; and (b) the mail versus the telephone intervention conditions. We ran separate models for each predictor/outcome combination: the linear slope, both linear and quadratic trends, and a third that included linear, quadratic, and cubic trends. The linear trend indicates the direction and rate of change, while the quadratic and cubic trends indicate how the rate of change increased or decreased at some point during the observation period. We report results from the best fitting of these three models for each predictor/outcome combination. All tables and figures present observed unimputed data.

For health care utilization data, we employed chi-square analyses to compare proportions of participants in each condition who had at least one instance of each type of health care service. We then conducted logistic regression analyses predicting use of each type of health care service from study condition and baseline CES-D score.

## Results

### Recruitment, Randomization, and Follow-Up

Of 12051 total study recruitment brochures mailed to depressed and nondepressed HMO members, 291 participants (2.4%) entered confirmed HMO membership numbers at the study home page and proceeded to the online consent form and baseline assessment battery. Subsequently, 255 members (87.6%) were automatically randomized by the website (using random sequence software) to one of three groups: 100 to the TAU control group, 75 to the ODIN intervention with postcard reminders group, and 80 to the ODIN intervention with telephone reminders group.

Fifty-five of the 255 enrolled participants were from the nondepressed recruitment group (0.9% of those invited), and 200 were from the depressed recruitment group (3.3% of those invited). The randomized sample was more likely to be female (77% vs 71% of the non randomized sample, *P* = .03) and older (64% were 45 years or older vs 52% of the non randomized sample, *P* < .001).

Follow-up completion rates for all groups combined were 64% (n = 164) at 5 weeks, 68% (n = 173) at 10 weeks, and 66% (n = 169) at 16 weeks. Overall, 209 participants (82%) completed at least one post-baseline assessment. Compared to participants completing at least one follow-up (baseline CES-D mean = 28.9, SD = 13.0), subjects who were lost to follow-up had higher baseline CES-D scores (mean = 33.3, SD = 12.6; *t* = 2.08, *P* = .04) and were slightly older (average age 47.7 vs 42.9, *P* = .006), but they did not differ with respect to gender (*P* = .08). Participants in the control condition were more likely to have completed at least one follow-up assessment (93%) than participants in either the telephone reminder intervention (76%) or the mail reminder intervention conditions (73%, *P* = .001).

### Comparability of Conditions

Table 1 presents the frequency of participant log-ons for the mail and telephone reminder intervention conditions and the same data from our earlier randomized trial [11]. Participants in the two intervention groups with different reminder modes did not differ in the number of log-ons to the website (*t* = .45, *P* = .65), but both groups together did access the website significantly more often (*t* = 5.74, *P* < .001) than participants in our initial study [11], which was nearly identical in design except for the lack of reminders.

Study conditions did not differ with respect to recruitment group, gender, or baseline CES-D and SF-12 scores; however, participants in the control group were more likely to be college graduates and were significantly older (Table 2).

**Table 1:** Frequency of ODIN website usage for mail and telephone reminder participants, and participants from the 1999 study [11] with no reminders

	Mode (Modal Frequency*)	**Median**	**Mean (SD)**	**Range**
Mail reminder	1 (28%)	4	5.9 (6.2)	1–33
Telephone reminder	1 (25%)	3	5.6 (5.8)	1–27
No reminder†	1 (41%)	2	2.6 (2.5)	1–20

^*^ The modal frequency is the percent of participants in each condition who had the modal (most frequent) number of log-ons, which was 1.

^†^ From the initial ODIN study [11], with no reminders to use the Internet site

**Table 2:** Comparison of experimental condition on baseline demographics

	**ODIN Group with** **Mail Reminder (n = 75)**		**ODIN Group with** **Phone Reminder (n = 80)**		**Control(n = 100)**		Significance*
	**Mean**	**SD**	**Mean**	**SD**	**Mean**	**SD**	
Age	50.3	10.8	44.4	10.5	45.0	10.6	< .001
Female	72.0%		83.8%		76.0%		.20
Minority	6.7%		5.1%		6.0%		.91
Married	49.3%		60.3%		67.0%		.06
College graduate	38.7%		38.0%		56.0%		.02
“Depressed” at case-finding	72.0%		83.8%		79.0%		.20

^*^ Participant age was compared with an ANOVA. All other comparisons were made with chi-square analyses.

### Depression

Figure 2 shows that participants in the intervention conditions improved more than those in the control group on self-reported depression (F_1,523_ = 4.93, *P* = .03 for the linear slope), with an estimated difference in effect size of 0.277 standard deviation units. The graph displays the group means for each participant's change in CES-D from their baseline score, across all assessment points. The random effect regression parameter estimate was 0.25 (95% CI = 0.03–0.58). This effect held up even when controlling for baseline differences in age and education. We did not find any difference between the two treatment conditions.

We tested clinical significance [28] by examining how many cases moved over time from the “disordered” to the “non disordered” CES-D ranges. The CES-D has two cutoff scores: a score of ≥ 16 is considered “moderately depressed,” and a score of ≥ 28 is considered “severely depressed” [12,29]. We compared the intervention conditions (combined) against the control condition using these categories. A total of 211 participants were above the lower of the two CES-D cutoff scores (≥ 16) at baseline (75 control and 136 treatment). Of these moderately depressed participants, 137 completed the 16-week follow-up. At that final follow-up, 56% (n = 42/75) of these participants in the treatment group were still in the moderately depressed range, compared to 76% (n = 47/62) of the control sample (chi^2^ = 5.8, *P* = .02).

We also examined the 149 participants who were above the severely depressed cutscore (CES-D ≥ 28) at baseline (53 control and 96 treatment). Of these, 93 participants completed the 16-week assessment; 42% (n = 20/48) of the intervention cases were still in the severely depressed range at this final follow-up, compared to 62% (n = 28/45) of the control cases (chi^2^ = 3.9, *P* = .05). Using either moderate or severely depressed scoring criteria, significantly more treatment participants (20%) moved from the clinical to normal range by the end of the study.

Because control participants were more likely to have completed at least one follow-up assessment than intervention participants, we examined whether the significant outcome results may have been a function of bias in the followed sample. This is a consideration because random effects regression methods yield unbiased estimates of missing follow-up data only if the missingness is ignorable (ie, can be predicted from patient characteristics and is unrelated to the study outcome). If loss to follow-up is a function of study outcome, the analyses conducted with imputed but possibly biased data may not accurately reflect the true outcomes. Therefore, we ran a repeated measures analysis predicting follow-up completion at each time point from baseline depression severity, age, sex, recruitment group, and educational attainment. In this model, younger age, male gender, and ODIN intervention assignment all increased the likelihood of missing a follow-up assessment. None of these factors predicted treatment outcome, suggesting they would be unlikely to contribute to the treatment outcomes that we found. However, it is not possible to completely prove that imputed follow-up data are unbiased. Therefore, our results clearly need replication in a sample with minimal and nonsystematic attrition.

**Figure 2:**
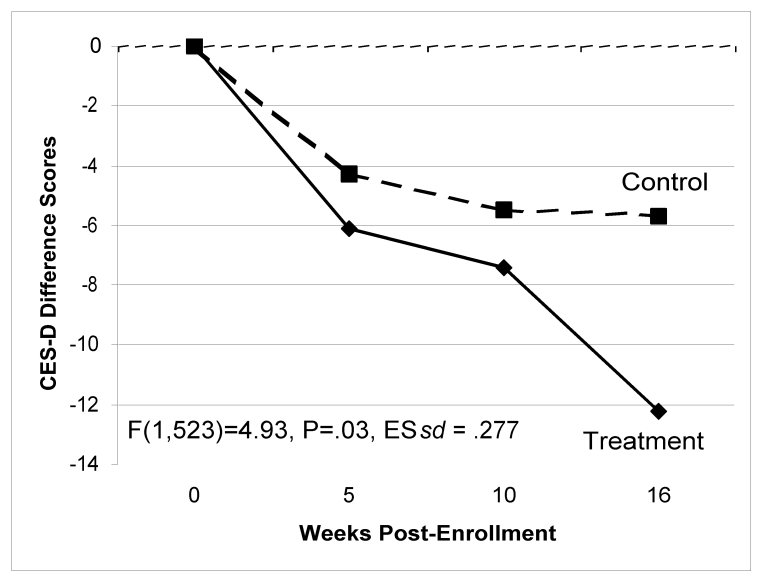
CES-D scores over time by condition (both treatment conditions combined)

**Table 3:** Self-reported depression outcomes (CES-D) for the total sample and selected subsamples

			**Baseline**		**5-Week**		**10-Week**		**16-Week**		
**Group**	**Study Condition**	**N**	**Mean**	**SD**	**Mean**	**SD**	**Mean**	**SD**	**Mean**	**SD**	*P* value*
**Total Sample**	Mail reminder	75	30.3	11.9	23.0	10.8	21.7	12.4	18.2	12.8	.03
	Phone reminder	80	31.3	13.2	26.3	13.3	24.9	13.1	19.0	13.1	
	Control	100	28.0	13.6	23.7	12.9	22.5	13.1	22.3	13.8	
**Depression Recruitment Cases**	Mail reminder	54	31.4	11.8	24.7	11.6	22.3	12.9	18.5	13.1	.08
	Phone reminder	67	31.3	13.4	24.8	13.3	24.4	13.2	20.0	13.8	
	Control	79	28.8	13.6	23.0	12.8	22.6	12.7	22.9	13.8	
**High Baseline CES-D**	Mail Reminder	58	35.2	8.4	26.5	10.0	25.3	11.8	19.7	12.3	.02
	Phone reminder	64	36.2	9.2	29.8	12.3	28.6	11.7	20.1	12.1	
	Control	69	35.4	9.1	28.1	12.0	26.1	12.6	26.7	13.1	
**Female**	Mail reminder	54	31.3	12.3	23.5	11.1	21.6	12.6	17.8	13.4	.17
	Phone reminder	67	30.1	13.5	26.7	14.0	24.8	13.2	20.0	13.4	
	Control	76	28.9	13.4	24.2	12.6	22.6	13.5	22.3	14.0	

*P* value for the random effects regression comparing two treatment conditions combined vs the control condition

Table 3 presents depression results for several subgroups, to generate hypotheses for future studies. We limited these exploratory analyses to subgroups with larger samples. These included female participants (n = 197; linear model fit best, time × treatment F_1,419_ = 1.92, *P* = .17); participants with higher baseline CES-D scores (CES-D > 20; n = 191; quadratic model fit best, time × time × treatment F_1,381_ = 5.14, *P* = .02; effect size = 0.537 standard deviation units); and participants recruited from among HMO members with depression diagnoses in their medical records (n = 200; linear model fit best, time × treatment F_1,403_ = 3.09, *P* = .08).

### Functioning

We did not find any statistically significant intervention effects on the physical components (PCS) or mental components (MCS) subscales of the SF-12 (Table 4).

**Table 4:** Self-reported SF-12 physical components scale (PCS) and mental components scale (MCS) for the total sample

			**Baseline**		**5-Week**		**10-Week**		**16-Week**		
**Subscale**	**Study Condition**	**N**	**Mean**	**SD**	**Mean**	**SD**	**Mean**	**SD**	**Mean**	**SD**	Significance*
PCS	Mail reminder	75	46.5	7.2	45.0	7.6	47.2	7.1	47.2	7.7	.92
	Phone reminder	80	44.9	7.5	45.4	6.9	45.9	6.9	47.3	6.9	
	Control	100	45.0	7.7	45.5	6.8	45.0	7.3	46.3	7.4	
MCS	Mail reminder	75	34.5	6.9	33.7	7.5	34.9	6.9	34.7	8.9	.18
	Phone reminder	80	35.3	9.1	35.1	7.0	33.7	7.8	32.3	7.6	
	Control	100	34.4	7.2	34.8	7.8	34.4	8.0	35.5	8.8	

^*^ The significance column displays the alpha for the random effects regression analyses comparing the two intervention conditions combined vs the control condition.

### Dose-Adjusted Effects

We failed to find statistically significant interactions between the total number of ODIN sign-ins (our measure of dose) and CES-D or SF-12 outcomes (data not shown).

### Health Care Utilization

In the 12 months following randomization, we found no differences in the use of mental health or general medical services or psychoactive medications across all conditions (Table 5).

**Table 5:** Health care services in the 12 months post-randomization, by study condition

	**TAU Condition(n = 100)**			**Mail Reminder Condition** **(n = 75)**			**Telephone Reminder** **Condition(n = 80)**			
	**Mean**	**SD**	N (%)> 0*	**Mean**	**SD**	**N (%)> 0**	**Mean**	**SD**	**N (%)> 0**	**Significance^†^**
**Mental Health Services**										
Outpatient visits	4.2	7.2	49 (49%)	3.1	5.7	35 (47%)	6.2	9.3	46 (57%)	.61
Total Rx dispenses	8.1	7.4	85 (85%)	7.7	6.2	68 (91%)	9.5	7.9	72 (90%)	.20
TCA dispenses	0.8	3.2	14 (14%)	0.3	1.4	7 (9%)	0.4	1.5	9 (11%)	.37
SSRI dispenses	4.0	4.1	65 (65%)	3.1	3.3	46 (60%)	3.4	3.7	46 (61%)	.48
Any MH Service			90 (90%)			71 (95%)			75 (94%)	
**General Health Services**										
Outpatient visits	9.7	9.5	95 (95%)	7.8	7.2	78 (97%)	8.5	7.1	73 (97%)	.31

Rx = any prescribed medication; TCA = tricyclic antidepressant; SSRI = selective serotonergic reuptake inhibitor antidepressant; MH = mental health

^*^ N (%) > 0 is the number and percent of the sample that had at least some level (> 0) of each type of treatment service.

^†^ The significance column displays the alpha for chi-square analyses comparing the proportion with *any* of each type of health care services, for the two intervention conditions combined vs the control condition.

## Discussion

We detected a modest but statistically and clinically significant advantage for the two treatment conditions relative to the control group on self-reported depression, but not on functioning. To the best of our knowledge, this study is the first to find significant effects for a pure self-help or “unattended” Internet program, where the intervention was delivered without any adjunct person-to-person contact.

This study is also the first to find Internet intervention effects in the context of a TAU control condition. TAU was essentially another potentially active treatment, with 93% of participants receiving at least some traditional mental health care in the year following randomization (84% through the week 16 follow-up), the majority of which was antidepressant medication. This high background level of depression treatment and other health care had the potential to obscure differences between conditions. Nonetheless, we still observed an advantage for the ODIN intervention.

While the magnitude of this outcome was relatively modest, it compares favorably with other traditional, stand alone bibliotherapy interventions such as self-help books [3]. More importantly, the potential public health implications of these findings are considerable. The low incremental costs of delivering this Internet program makes it feasible to offer this or similar programs to very large populations (health plans, large employer groups, universities) where Internet access is widespread. Interventions with a small average effect may have substantial public health impact when applied to a large number of people, as a modest but meaningful number of patients will not develop the target disorder as a result of this small, average improvement [30].

Is the observed effect size of 0.277 standard deviation units (0.537 in cases with higher baseline depression) of sufficient magnitude to merit much enthusiasm? In meta-analyses of depression evidence-based psychotherapy *efficacy* randomized controlled trials (where the control condition is typically an easily surmounted no treatment or waitlist control), the difference in effect size is typically much higher, averaging around 1.56 standard deviation units [31]. However, when (as in this randomized controlled trial) the evidence-based psychotherapy is provided in the context of TAU [32], this effect size advantage typically shrinks substantially. Gaffan [31] and Gloaguen [33] find only small to medium mean effect sizes favoring CBT when it is compared to behavioral therapy (0.27), “other” psychotherapy (0.23), or pharmacotherapy (0.27). In this context, our TAU control condition is best thought of as a blend of evidence-based and non-evidence-based psychosocial and pharmacotherapy treatments [34]. Therefore, the observed effect size of 0.277 standard deviation units is roughly consistent with the effect sizes of this meta-analysis when traditional, face-to-face CBT is compared to these other treatments.

The mail and telephone reminders similarly increased the frequency of visits to the ODIN site, relative to our first study with no reminders [11]. We are therefore inclined to use postcard reminders in the future because they are much less costly than telephone reminders.

Our failure to detect effects on health care utilization was not unexpected. A follow-up period of two years or more is typically needed to detect impacts of an intervention on health care utilization [35]. Further, because health care utilization typically has very high variance (a small number of patients use an extreme amount of health care), very large samples are typically needed for adequate power [36].

### Limitations

This study had several limitations. First, despite providing gift certificates for completed assessments, follow-up rates averaged around 66%—although 82% of participants completed at least one follow-up assessment. These rates are comparable to the follow-up rates obtained in our earlier study [11] and are similar to, if not better than, rates seen in other Internet intervention trials (reviewed by Eysenbach [37]).

Second, subjects lost to follow-up were slightly more depressed, slightly older, and less likely to be in the control group. All these factors, but particularly the interaction between experimental condition and attrition, limit our confidence in our results, although post-hoc analyses suggest that confounding effects were unlikely to have accounted for the observed results.

Our enrollment rates were also quite low, with 3.3% of the “depressed” recruitment sample and 0.9% of the “nondepressed” recruitment sample enrolling in the study, respectively. We have no information on why so many declined to enroll. Because the majority of the “depressed” recruitment sample was receiving traditional depression care (all had depression diagnoses in their medical charts), perhaps they felt no need to augment their traditional care with our self-help program. Among the nominally “nondepressed” recruitment sample, we had hoped to enroll previously unrecognized cases of depression [38]. However, the 1% “nondepressed” enrollment rate suggests that only a small minority of these undetected cases found our study of interest. Perhaps some of these individuals did not recognize their own depression and thus would not have seen the program as applicable. Still others may have been receiving other depression care outside of this HMO, which we could not know about from the HMO records. Regardless of the reasons for the low enrollment, these rates are not an indication of the *acceptability* of this intervention or any Internet program offered outside of a research trial. The unique features of randomized trials (a chance of being assigned to the no-access control group, repeated reminders to complete assessments over time, burdensome questionnaires) create barriers to participants that likely contribute to lower research enrollment rates, but which have no counterparts in usual clinical care implementation of these types of programs.

This study was also limited by its reliance on a single, self-reported measure of depression. We decided against using research diagnostic interviews because the accompanying in-person or telephone interview contacts had the potential to impart quasi-therapeutic benefits that, in turn, might have swamped the small benefit expected from the ODIN intervention. Further, the target population for the ODIN website includes persons who may have low level or subdiagnostic depression symptoms, as well as individuals who meet full diagnostic criteria for major depression or other DSM mood diagnoses. Relying on DSM mood diagnosis as a primary outcome might have missed the effects of the ODIN intervention on depression symptoms below the level of a full diagnosis.

Finally, our follow-up period of 16 weeks was extremely brief. We must examine this intervention's longer term impacts on depression, health care utilization, and quality of life. Future studies should include a much longer follow-up and a broader range of assessment domains.

### Conclusions

The lessons we have learned from this investigation are guiding our development of a completely new Internet intervention for depressed young adults. This new program emphasizes behavioral activation, or increasing pleasant activities, as the main therapeutic technique [39].

We are encouraged by the results of this study, while acknowledging the positive effects are modest in magnitude. Nonetheless, we view low intensity, widely available interventions as an important piece of an overall, population-based strategy for reducing depression disorder and symptomatology. The marginal costs of delivering this pure self-help Internet program to each additional individual are very minimal, given that there is no staff time associated with the delivery of the intervention content. Therefore, it is feasible to offer this type of program to entire populations where Internet access is widespread, such as universities and large employers.

## Figures and Tables

**Figure 2a:**
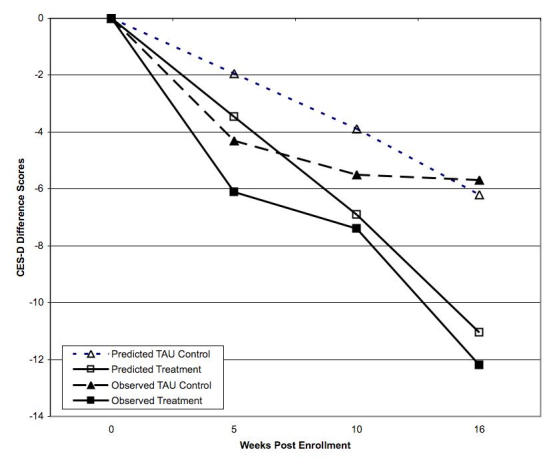
Predicted and observed data for the main outcome measure, the CES-D

## Appendix

Additional figure (complementing figure 2) showing both the predicted and observed data for the main outcome measure, the CES-D. (added 08 June 2005)

## Abbreviations

CES-D: Center for Epidemiological Studies Depression Scale
CBT: cognitive behavioral therapy
HMO: health maintenance organization
MCS: Mental Component Summary
ODIN: Overcoming Depression on the InterNet
PCS: Physical Component Summary
SF-12: Short Form 12
TAU: treatment as usual
